# Supplementary material for: LC-MS/MS analysis of lesional and normally looking psoriatic skin reveals significant changes in protein metabolism and RNA processing
Source: PLoS One. 2021 May 26;16(5):e0240956. doi: 10.1371/journal.pone.0240956 (PMC8153457; doi:10.1371/journal.pone.0240956)
Supplement: S1 Fig — In the figure, rows and columns refer to the individuals participated in LC-MS/MS study (Table 1). The entry in the i-th row and j-th column is the Pearson correlation rij of protein expression profiles of skin donors’ i and j. The color scale at the bottom indicates the correlation strength. (PDF) [file pone.0240956.s001.pdf]

Supplementary methods

To assess variability in individual samples and groups of samples, and define how protein expression profiles could be influenced by the donors' age, we generated a color-coded matrix of the Pearson correlation coefficients (**Figure S1**). Probe sets with normalized signals were used to calculate the correlations between 15 protein arrays representing uninvolved and lesional skin of psoriasis patients and the skin of healthy volunteers. Each row and column corresponded to an individual and the entry in the i-th row and j-th column was the Pearson correlation r_{ij} between the protein expression profiles of skin donors i and j. The data on skin donors were taken from **Table 1**.

Supplementary analysis

We evaluated variations in protein expression profiles among the individuals of different age participated in our LC-MS/MS study. A color-coded pairwise correlation matrix is shown in **Figure S1**. Since samples were obtained from the same tissue and equally processed using the same techniques, the generated protein expression profiles showed strong correlations to each other. The Pearson correlation coefficient ranged from 0.811 to 1. As expected, higher correlation was observed among samples of the same type. For the samples obtained from healthy skin, the correlation coefficient ranged from 0.871 to 0.988. For the samples obtained from uninvolved skin, the correlation coefficient ranged from 0.981 to 0.997. For the samples obtained from lesional skin, the correlation coefficient ranged from 0.946 to 0.993.

As we noticed, the protein expression profiles of participant 10 (79 y.o.) and her fellow groupmates had less correlation to each other (**Figure S1, row 10, columns 6-10**). After examining medical history of participant 10 (**Table 1**), we proposed that this irregularity might not be primarily explained by her age, because one of her groupmates, namely participant 9, who was 77 y.o., and belonged to the same age group, was in line with the others (0.893). Thus, due to participant 10 was diagnosed with several medical conditions; we concluded that at least one of these conditions could cause the changes in her protein expression profile.

In the same time, the protein expression profiles of participant 10 and psoriasis patients (**Figure S1, row 10, columns 1u-5u and 1l-5l**) did not show higher correlations compared to ones of participant 10 and healthy volunteers (**Figure S1, row 10, columns 6-10**). For the samples of *uninvolved* skin (**Figure S1**, **row 10**, **columns 1u-5u**), the three lowest correlations (0.845, 0.868 and 0.869) did not exceed 0.871, which was the lowest correlation of participant 10 with her fellow groupmates (**Figure S1**, **row 10**, **column 8**). Moreover, the highest correlation - 0.879 (**Figure S1, row 10, column 2u**) was less than three highest correlations in the same group (**Figure S1, row 10, columns 6-10**). In turn, when we compared the correlations of participant 10 and other volunteers (**Figure S1**, **row 10**, **columns 6-9**) with correlations of participant 10 and uninvolved skin of psoriasis patients (**Figure S1**, **row 10**, **columns 1u-5u**) using Mann-Whitney U-test, the test did not reveal significant changes of correlation coefficient ($\alpha = 0.05$).

For the samples of *lesional* skin (**Figure S1**, **row 10**, **columns 1l-5l**), all correlations did not exceed 0.871 – the lowest correlation of participant 10 with her fellow groupmates (**Figure S1**, **row 10**, **column 8**). Moreover, when we compared the correlations of participant 10 and other volunteers (**Figure S1**, **row 10**, **columns 6-9**) with correlations of participant 10 and samples of lesional skin (**Figure S1**, **row 10**, **columns 1l-**

5l) using Mann-Whitney U-test, the test results suggested significant changes of correlation coefficient (α=0.05).

Thus, we found that correlation profile of participant 10 was similar to other profiles of healthy volunteers participated in our study (**Figure S1, rows 6-10**). Particularly, her skin sample showed the highest correlations with other samples of healthy skin (**Figure S1, row 10, columns 6-9**). In turn, it showed less correlation with the samples of uninvolved skin (**Figure S1, row 10, columns 1u-5u**) although we could not confirm significance of the observed differences using the statistical analysis. In addition, it had the lowest correlations with the samples of lesional skin (**Figure S1, row 10, columns 1l-5l**) and changes in correlation coefficient were statistically significant compared to healthy skin (**Figure S1, row 10, columns 6-9**).

In the group of psoriasis patients, participant 5 showed higher correlations with tested samples of uninvolved skin (**Figure S1**, **row 5l**, **columns 1u-5u**) compared to his groupmates (**Figure S1**, **rows 1l-5l**, **columns 1u-5u**). Moreover, same sample had higher correlations with samples of healthy skin (**Figure S1**, **rows 1l-5l**, **columns 6-10**). In the first case, non-parametric Mann-Whitney U-test (α = 0.05) revealed that changes in correlation coefficient were significant. In the second case, the differences were not significant (α = 0.05). Respectively, we proposed that two parameters, namely his medical condition(s) and low PASI (**Table 1**) might contribute to irregularities in the patient's 5 protein expression profile.

S1 Fig

Skin	Uninvolved skin					Lesional skin					Healthy skin					Age
samples	1u	2u	3u	4u	5u	1l	2l	3l	4l	5l	6	7	8	9	10	
1u	1	0.997	0.987	0.995	0.986	0.900	0.913	0.907	0.928	0.979	0.920	0.955	0.933	0.967	0.868	30
2u	0.997	1	0.988	0.995	0.983	0.886	0.906	0.904	0.920	0.972	0.915	0.959	0.938	0.971	0.879	40
3u	0.987	0.988	1	0.985	0.988	0.889	0.910	0.901	0.927	0.970	0.926	0.959	0.962	0.985	0.873	48
4u	0.995	0.995	0.985	1	0.981	0.880	0.905	0.889	0.921	0.972	0.900	0.941	0.926	0.962	0.845	60
5u	0.986	0.983	0.988	0.981	1	0.916	0.920	0.906	0.940	0.983	0.946	0.967	0.961	0.979	0.869	68
1l	0.900	0.886	0.889	0.880	0.916	1	0.979	0.963	0.979	0.959	0.881	0.884	0.851	0.868	0.811	
2l	0.913	0.906	0.910	0.905	0.920	0.979	1	0.974	0.993	0.963	0.851	0.882	0.866	0.889	0.826	
3l	0.907	0.904	0.901	0.889	0.906	0.963	0.974	1	0.974	0.946	0.843	0.890	0.850	0.877	0.870	
4l	0.928	0.920	0.927	0.921	0.940	0.979	0.993	0.974	1	0.972	0.871	0.899	0.885	0.906	0.829	
5l	0.979	0.972	0.970	0.972	0.983	0.959	0.963	0.946	0.972	1	0.919	0.939	0.919	0.947	0.848	
6	0.920	0.915	0.926	0.900	0.946	0.881	0.851	0.843	0.871	0.919	1	0.971	0.934	0.935	0.880	39
7	0.955	0.959	0.959	0.941	0.967	0.884	0.882	0.890	0.899	0.939	0.971	1	0.955	0.967	0.935	53
8	0.933	0.938	0.962	0.926	0.961	0.851	0.866	0.850	0.885	0.919	0.934	0.955	1	0.988	0.871	60
9	0.967	0.971	0.985	0.962	0.979	0.868	0.889	0.877	0.906	0.947	0.935	0.967	0.988	1	0.893	77
10	0.868	0.879	0.873	0.845	0.869	0.811	0.826	0.870	0.829	0.848	0.880	0.935	0.871	0.893	1	79

0.81 1.00

S1 Fig. Analysis of data variability in the samples of healthy, uninvolved and lesional skin. In the figure, rows and columns refer to the individuals participated in LC-MS/MS study (Table 1). The entry in the i-th row and j-th column is the Pearson correlation r_{ij} of protein expression profiles of skin donors' i and j. The color scale at the bottom indicates the correlation strength.
