# Supplementary material for: LC-MS/MS analysis of lesional and normally looking psoriatic skin reveals significant changes in protein metabolism and RNA processing
Source: PLoS One. 2021 May 26;16(5):e0240956. doi: 10.1371/journal.pone.0240956 (PMC8153457; doi:10.1371/journal.pone.0240956)
Supplement: S2 Fig — (PDF) [file pone.0240956.s002.pdf]

Supplementary methods

To validate the data of LC-MS/MS study, we analyzed the obtained samples of lesional skin of psoriasis patients and skin of healthy volunteers for the presence of selected antigens, namely TYMP, S100A2 and NYMPT, using the method of immunohistochemistry. Briefly, skin samples were fixed in formalin and embedded in paraffin. The prepared paraffin blocks were cut to obtain 5 µm thick sections. Prior the experiment, the sections were deparaffinized in xylene (2x 10 min) and rehydrated consequently in 100%, 95%, 70% ethanol (v/v) and distilled water. After blocking the endogenous peroxidases (1% H_2O_2; 30 min), and sites of non-specific binding (5% donkey serum, 1% BSA, 0.2% Triton X-100, 0.05% Tween 20 in PBS; 30 min), the slides were incubated overnight at 4°C with one of the following primary antibodies: NAMPT (NB100-594); TYMP (NBP1-84916) or S100A2 (27540002). The primary antibodies were purchased from Novus Biologicals (USA). After washing with TBST (3 x 15 min), the slides were incubated with polymer-conjugated secondary antibodies (ImmPRESS® Horse Anti-Rabbit IgG PLUS Polymer Kit, MP-7801, Vector Laboratories, USA) for 20 min at room temperature. Then, slides were washed again (2 x 5 min), stained with the substrate of horseradish peroxidase, ImmPACT® DAB EqV (SK-4103, Vector Laboratories) and counterstained with hematoxylin. The images were captured using Leica DM4000 B (Leica Microsystems, Germany) and analyzed using LAS AF imaging software, version 3.1.0, build 8587 (Leica Microsystems, Germany).

Supplementary analysis

The presence of selected DEPs, namely NAMPT, TYMP and S100A2 was confirmed in lesional skin using the method of immunohistochemistry with the specific antibodies (**Figure S2**, **upper row**). The mentioned DEPs exhibited similar staining patterns. The highest expression levels were observed in the basal layer, at the edges of rete ridges, presumably, at the places where immune cells traverse the basement membrane (**indicated by black arrow**). Then, they gradually declined until the upper spinous layer. NAMPT and S100A2 were predominantly located in the cytoplasm whereas TYMP was also found in the extracellular space. The named antigens were also present in infiltrated immune cells (**indicated by blue arrows**). In addition, NAMPT, TYMP and S100A2 were also detected in the stratum corneum, in the areas associated with parakeratosis (**marked by asterisks**). In the same time, we did not see any visible staining for the tested DEPs in the skin of healthy volunteers, despite the cell nuclei were strongly counterstained with hematoxylin (**Figure S2**, **lower row**).

S2 Fig

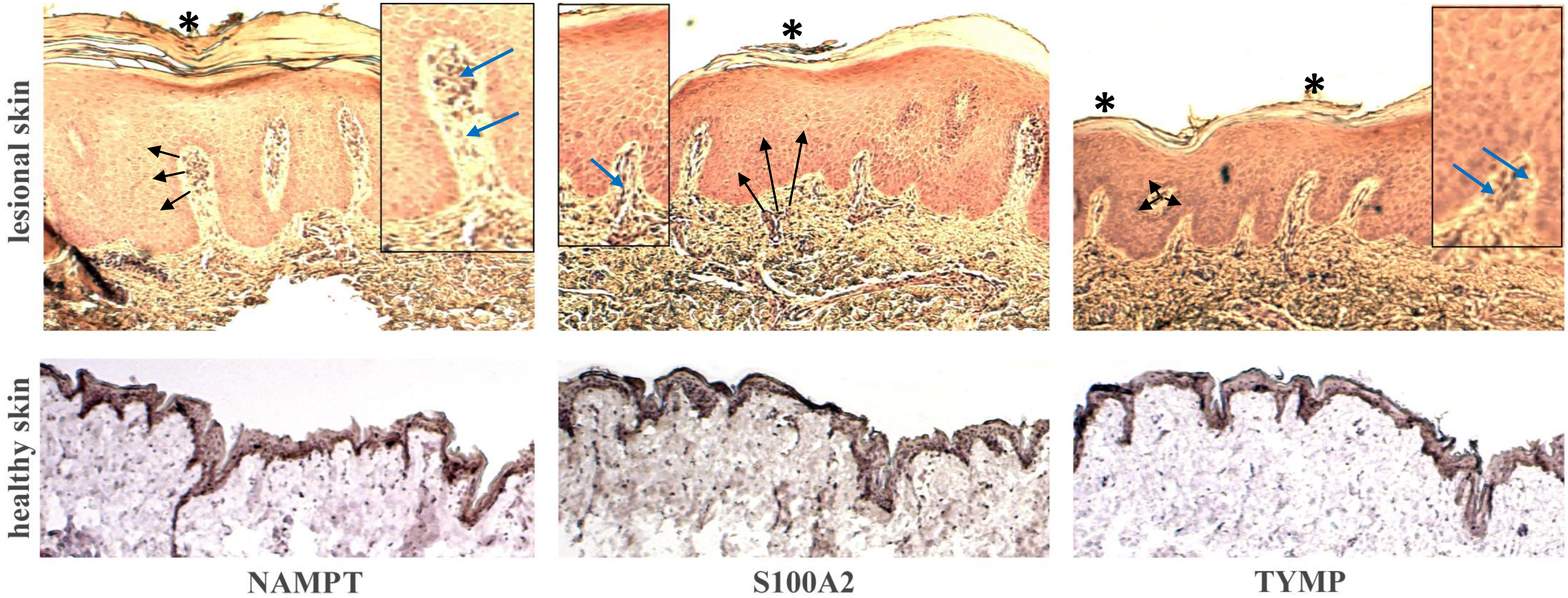

S2 Fig. **Immunohistochemical staining for NAMPT, S100A2 and TYMP in lesional psoriatic skin and skin of healthy volunteers**. The magnification of original images was set at x 100. Blue arrows indicate on immune cells stained for the specific antigen. Black arrows trace the proposed trajectories of the immune cells traversed the basement membrane. Asterisks indicate on parakeratotic areas of the stratum corneum stained positive for the specific antigens. Images located inside the black boxes are the selected areas of the histological sections at higher magnification.
